# Cellular mechanisms underlying adult tissue plasticity in *Drosophila*

**DOI:** 10.1080/19336934.2022.2066952

**Published:** 2022-04-26

**Authors:** Hiroki Nagai, Masayuki Miura, Yu-ichiro Nakajima

**Affiliations:** Graduate School of Pharmaceutical Sciences, the University of Tokyo, Tokyo, Japan

**Keywords:** Adult tissue plasticity, stem cell, dedifferentiation, polyploidy, *Drosophila melanogaster*, regeneration, nutrient response, midgut, brain, testes

## Abstract

Adult tissues in Metazoa dynamically remodel their structures in response to environmental challenges including sudden injury, pathogen infection, and nutritional fluctuation, while maintaining quiescence under homoeostatic conditions. This characteristic, hereafter referred to as adult tissue plasticity, can prevent tissue dysfunction and improve the fitness of organisms in continuous and/or severe change of environments. With its relatively simple tissue structures and genetic tools, studies using the fruit fly *Drosophila melanogaster* have provided insights into molecular mechanisms that control cellular responses, particularly during regeneration and nutrient adaptation. In this review, we present the current understanding of cellular mechanisms, stem cell proliferation, polyploidization, and cell fate plasticity, all of which enable adult tissue plasticity in various *Drosophila* adult organs including the midgut, the brain, and the gonad, and discuss the organismal strategy in response to environmental changes and future directions of the research.

## 1. Introduction

During the development of multicellular animals, tissues are formed through a dynamic process in which cells actively change their morphology and behaviours in concert with surrounding cells. In contrast, tissues in mature adults are quiescent in homoeostatic conditions. Nevertheless, mature adult tissues can flexibly remodel their structure upon environmental challenges, such as pathogenic infection and starvation. While pathogenic infection causes damages in host cells, tissues activate the turnover of damaged cells to regenerate the injured region. Similarly, while starvation causes tissue shrinkage, tissue size recovers after refeeding. These adaptive abilities at the tissue level in adults, termed adult tissue plasticity, are crucial to maintaining organismal health: failures in regenerative/nutrient responses lead to inflammatory diseases, cancers, and metabolic dysfunctions [1–3]. Therefore, understanding the mechanisms of adult tissue plasticity is an important issue in biological research as well as in basic medicine.

Tissues consist of various cell types including stem cells, progenitor cells, and differentiated cells, which together cooperate to ensure physiological functions. For instance, cells surrounding stem cells (stem cell niche) regulate proper stem cell division, which is essential for the turnover of old/damaged cells while maintaining epithelial barrier function [4]. The fruit fly *Drosophila melanogaster* serves as an ideal model organism to understand complex cellular mechanisms in adult tissue plasticity for the following reasons: (1) the plethora of extant genetic tools enables cell type-specific gene manipulations in which two distinct cell types can be independently modulated with different binary expression systems (e.g. GAL4/UAS and QF/QUAS), (2) whole-mount tissue can be histologically analysed at a single-cell level, even in *ex vivo* condition, and (3) cellular functions and lineage hierarchy are largely conserved from fly to mammals. Moreover, *Drosophila* adult tissues are highly plastic against environmental stresses while relatively quiescent under homoeostatic conditions. Recent studies using *Drosophila* have revealed the mechanisms of stem cell regulation, polyploidization in differentiated cells, and cell fate plasticity during environmental responses. Notably, these cellular processes are commonly utilized in various contexts, and consequently findings in *Drosophila* provide mechanistic insights into tissue plasticity in different animal species. In this review, we discuss the plasticity of *Drosophila* adult tissues in response to environmental stresses by highlighting recent reports in the adult midgut, the adult brain, and the adult gonad.

## 2. Adult tissue plasticity

### 2-1: Plasticity in the adult midgut during regeneration and nutrient fluctuation

**Figure 1.**
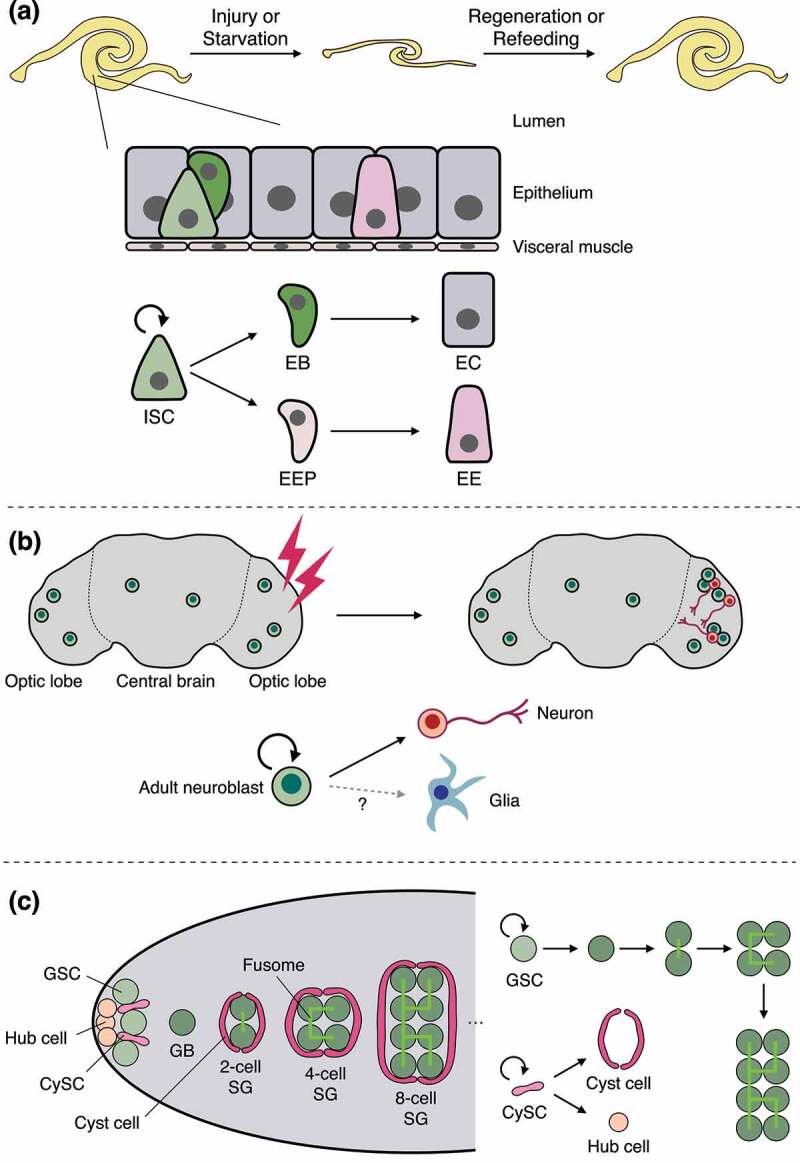
Cell lineage and plasticity in adult tissues.

As a primary site of host defence and absorption, the intestinal epithelium continuously interacts with orally ingested microorganisms and nutrients. The gut responds to environmental changes with cooperating functions of multiple intestinal cell types. Studies using the adult *Drosophila* midgut have contributed to unravelling such complex mechanisms due to the simplicity of fly tissue structure and cell lineage (Figure 1a). In the adult midgut, the multipotent intestinal stem cell (ISC) is the only cell type that exhibits mitotic activity and generates progenitor cells: enteroblasts (EBs) or enteroendocrine progenitors (EEPs), which terminally differentiate into absorptive enterocytes (ECs) or secretory enteroendocrine cells (EEs), respectively [5–9]. The cellular composition of the midgut is maintained in homoeostatic conditions but can be rearranged upon environmental challenges, driving adaptation of the intestinal tissue.

(a) Schematics of the adult midgut. The size of the adult midgut declines in response to injury or starvation but recovers during regeneration or upon refeeding. The midgut epithelium is a pseudostratified monolayer and covered by visceral muscle. The multipotent intestinal stem cell (ISC) generates an enteroblast (EB) or an enteroendocrine progenitor (EEP), which differentiates into an enterocyte (EC) or an enteroendocrine cell (EE). (b) Schematics of the adult brain. Recent studies have suggested the existence of adult neuroblasts both in the optic lobe and the central brain. Adult neuroblasts generate new neurons upon brain injury although their gliogenic capacity remains unclear. (c) Schematics of the adult male testis. Two types of stem cell exist in the male testis. One is the germline stem cell (GSC), which generates sperm cells through progressive differentiation of gonialblasts (GB) and spermatogonia (SG). The other is the somatic cyst stem cell (CySC), which generates cyst cells and hub cells. The hub cell maintains stemness of GSC and CySC. Cyst cells encapsulate GB and SG to support germline maturation into sperm cells. GB divides 4 times with incomplete cytokinesis, resulting in an SG cluster that is interconnected through a germline-specific organelle called the fusome (light green).

The organ size of the adult midgut is highly plastic to environmental changes; the adult midgut becomes bigger (grows) or smaller (shrinks) in size depending on context. For instance, the adult midgut shrinks acutely upon oral infection; however, the damaged midgut can regenerate after pathogen clearance [10–13]. Cell death in ECs, the largest and most abundant cell type in the midgut, is the principal cause of midgut shrinkage [14]. Remarkably, even when the midgut size decreases by 30–50% upon injury, it takes only 2–3 days to recover its original size [11,14], indicating that the adult midgut has a high regenerative capacity. In addition to gut-intrinsic regeneration mechanisms, extra-intestinal tissues, such as haemocytes and the trachea, also fuel the regenerative responses of the midgut [15–18]. Given that both haemocytes and trachea are allocated throughout the body, it is possible that similar inter-organ communications underly plasticity in other adult tissues.

In addition to injury, the organ size of the adult midgut dynamically changes in response to nutrient environments. In newly eclosed adults, the midgut grows rapidly during the first 4–7 days of adult life in a feeding-dependent manner [19–23]. Mating further promotes midgut growth by hormonal signalling [24–27], however, midgut size increases after eclosion even in virgin females [28], suggesting that a certain extent of organ growth is likely entirely nutrient dependent. In mature adults, starvation causes shrinkage of the midgut due to the combination of cell death in ECs, reduction in ISC numbers, and contraction of the visceral muscle [23,29–31]. The smaller midgut rapidly recovers after 7 days of refeeding. It is noteworthy that the two types of nutrient-driven midgut growth, one in response to first food intake after eclosion and the other during starvation-refeeding cycle, are regulated by common cellular mechanisms. These findings imply that the gut growth in the early adult is not merely a part of developmental process but rather can be considered a general response to nutrient fluctuation.

### 2-2. Plasticity in the adult brain during regeneration

The adult *Drosophila* brain, wherein tens of thousands of neurons form complex networks, governs sensory perceptions and processing against environmental changes. The optic lobe, a brain region connecting the compound eyes and the central brain, receives visual information from photoreceptor neurons and relays the information to the central brain where neural information is integrated and processed as memory, learning, or behaviour output [32]. Both brain regions consist of neuron and glia, the latter of which supports neuronal functions or constitutes the blood–brain barrier. The number of neurons is estimated to be ~100,000 in the optic lobe and ~50,000 in the central brain [33,34]. It is just beginning to be understood how such complex organ structures are maintained against environmental stresses (Figure 1b).

Recent studies have revealed a surprising regenerative potential in the adult brain [35–37]. A stab injury in which a thin metal needle is inserted into the head distorts brain structure and severely impairs locomotor activity. Nevertheless, both the optic lobe [36,37] and the central brain [35] are morphologically regenerated within 2 weeks of injury. This organ repair accompanies restoration of locomotor function, suggesting that the regenerated brain functions properly [35]. These results indicate that even the adult brain, where cellular turnover is normally much slower compared to the adult midgut, exhibits high plasticity in response to severe damage.

### 2-3. Plasticity in the male testis during nutrient fluctuation

The adult gonad constantly produces gametes for reproduction, which is essential for maintaining the species. To ensure proper gametogenesis, gonadal cellular arrangement is well organized in both mammals and *Drosophila* [38–40]. In the *Drosophila* male testis, germline stem cells (GSCs) are located at the apical tip of the tissue, surrounded by niche cells, called hub cells (Figure 1c). The hub cells maintain the stemness of adjacent GSCs, enabling an asymmetric outcome of GSC division in which one daughter cell initiates the differentiation program after leaving the niche. Besides GSCs, somatic cyst stem cells (CySCs) also reside in the apical tip of testis and support germline differentiation by producing cyst cells, which encapsulate differentiating GSC daughters. The differentiation of cyst cells is coordinated with GSC differentiation to ensure successful gamete production [41]. Similar to the male testis, in the female ovary, GSCs are maintained by niche cells (cap cells), and GSC differentiation is supported by escort cells and follicle cells, the latter of which are generated from somatic follicle stem cells [38].

Gametogenesis is an energy-consuming but indispensable process for species survival, despite not being necessary for the survival of individuals. To balance individual fitness and reproduction, gamete production is suppressed under starved conditions, but reactivated after refeeding [42–44]. At the cellular level, the number of GSCs in the adult male testis decreases during chronic nutrient stress and recovers in response to refeeding [30,31,44,45]. Defects in GSC recovery lead to shrinkage of the male testis and failure in spermatogenesis after repeated cycles of starvation and refeeding [45]. These findings suggest that regulation of the GSC population is a crucial component of testis plasticity against nutrient environment.

## 3. Regulation of stem cell activity

Proliferation of tissue stem cells is essential and an evolutionarily conserved process for cell turnover in adult tissues [46–48]. The activity of stem cells must be adapted to tissue demand because both excess and insufficient proliferation cause disruption of tissue homoeostasis. To direct appropriate stem cell behaviour, stem cell niches sense tissue conditions (e.g. harmful injury, scarcity of nutrients, or refeeding) and send signals to modulate the mitotic activity of stem cells.

### 3-1. Regulation of stem cell activity during regeneration after tissue damage

**Figure 2.**
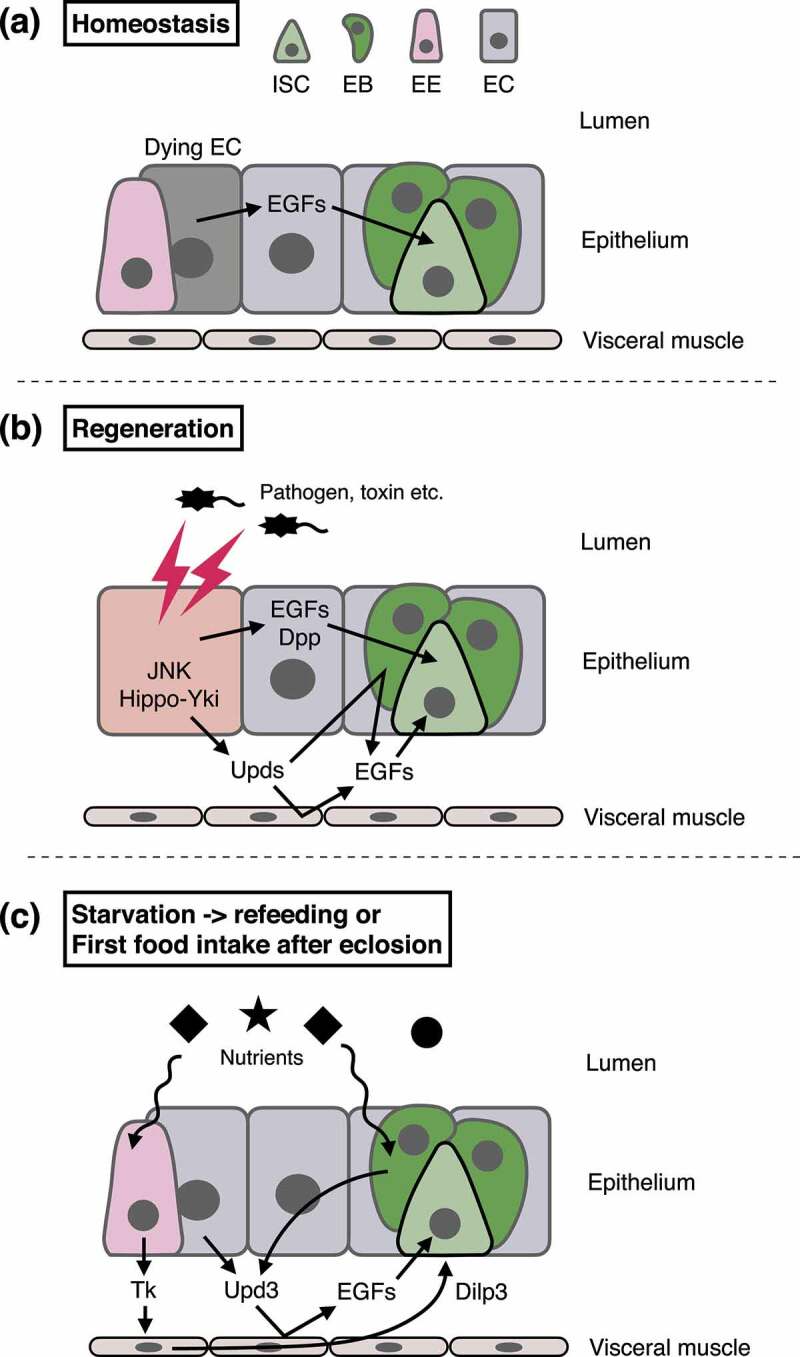
Regulation of ISC proliferation.

How does the stem cell niche switch stem cell activity from homoeostatic to regenerative? Numerous studies using the *Drosophila* adult midgut have addressed this question and provided mechanistic insights into the regeneration-specific activity of stem cells. In homoeostatic conditions, ECs undergoing apoptotic cell death secrete epidermal growth factors (EGFs) to promote the division of neighbouring ISCs (Figure 2a) [49,50] and the survival of EBs [51]. During regeneration, damaged ECs activate JNK signalling and Hippo-Yki pathway to produce unpaired (Upd) family (Figure 2b) [14,52–56]. Since Upds act on EB and visceral muscle to upregulate EGF production, the damage signal is amplified and widely spread from the injury site [50,57,58]. In addition to Upds, damaged ECs also secrete EGFs [11,49] and Dpp [59] to activate ISCs. These multiple cytokines, especially Upds, strongly stimulate ISC proliferation during regeneration. To suppress the production of Upds in homoeostatic conditions, autophagy negatively regulates JNK signalling [60] and the Hippo-Yki pathway [61], functioning as a gatekeeper in ECs. These findings have revealed the niche mechanisms by which the mitotic activity of ISCs is flexibly modulated in response to tissue damage.

(a) In homoeostatic conditions, dying ECs, but not healthy ECs, secrete EGFs to stimulate ISC division. (b) Upon midgut injury, damaged ECs activate JNK signalling and the Hippo-Yki pathway to upregulate *upd* cytokines, which induce EGF secretion from EBs and visceral muscle. Furthermore, damaged ECs also secrete EGFs and Dpp. These multiple cytokines and secreted factors strongly activate ISC proliferation. (c) Upon refeeding after starvation, ISC division is induced by Dilp3 secreted from visceral muscle and by Upd3 secreted from ECs and mechanosensitive EBs. EEs secrete the neuropeptide Tachykinin to increase Dilp3 production in visceral muscle.

The adult brain also relies on mitotic proliferation for repairing tissue damage despite having few to no active stem cells in homoeostatic conditions. While *Drosophila* neural stem cells, or neuroblasts, undergo apoptosis or cell cycle exit during the pupal stage [62–64], recent studies using the advanced lineage tracing approach have identified a rare cell population with neuroblast markers Deadpan (Dpn) in the adult brain [35,36,65]. Furthermore, miR-31a+ cells are identified as glial-neural bipotent progenitors in the adult brain [66]. During regeneration, Dpn+ cells enter the mitotic cell cycle and new Elav+ daughter cells are generated from Dpn-lineage. These results suggest that the Dpn+ cells are adult neuroblasts with neurogenic potential [35,36].

Unlike their neurogenic potential, the gliogenic capacity of adult Dpn+ cells remains controversial. New glial cells are generated in the central brain following injury despite the complete lack of gliogenesis in the damaged optic lobe. These regional differences in gliogenesis are accompanied by differences in neuroblast markers: while the optic lobe expresses only Dpn, the central brain expresses Dpn as well as Asense, Miranda, Inscuteable, and Earmuff [35,36,65]. These multiple neuroblast markers may underlie the gliogenic capacity of the central brain. Alternatively, glia themselves may generate new glia, as mitotic glial cells are observed in response to injury [35].

How is the quiescent adult neuroblast activated following injury? dMyc is up-regulated in brain cells around injury sites, and its overexpression is sufficient to activate Dpn+ cells [36]. Given that Myc can improve cellular fitness both in flies and mammals [67–70], it is noteworthy that neurogenesis after brain injury is followed by apoptosis in older neurons as a consequence of intercellular fitness comparison via isoforms of Flower [37,71]. These reports suggest the possibility that dMyc-mediated activation of quiescent adult neuroblasts contributes to neurogenesis after traumatic injury and the subsequent elimination of less fit neurons. Besides dMyc, the JNK ligand Eiger (Egr) may act as a niche factor regulating the adult neurogenesis. Egr is expressed in the adult brain and promotes the expansion of glioblastoma at least in the larval brain [72,73]. Moreover, JNK signalling is activated following brain injury independent of apoptosis [37]. Teasing out the roles of dMyc and Egr would be a fruitful way to advance our understanding of adult brain neurogenesis.

Does the adult brain exhibit a neurogenic potential throughout organismal life? The first 5 days in the adult stage are a critical period when the size of adult brain increases in response to physiological stimuli, such as visual information and social interactions [74–78]. Concordantly, Li et al. found that overexpression of Toll-2 during the critical period increases brain size via Yki-dependent cell proliferation [65]. In addition, brains in 1-day old adults exhibit higher mitotic activity than those in older adults in response to stab injuries [35]. These results support the notion that the adult brain is more plastic during the critical period. Intriguingly, however, mitotic events are detected even when stab injuries are performed 7 or 14 days after eclosion [35,36]. Moreover, Dpn+ adult neuroblasts are observed in the uninjured brain [36,65]. These observations suggest that the adult brain may maintain the neurogenic potential beyond the critical period and enable neurogenesis in response to severe brain injury.

### 3-2. Regulation of stem cell activity during nutrient fluctuation

Regulation of ISC proliferation contributes to the nutrient adaptation of the midgut as well as regeneration. Because active cell turnover in the adult midgut requires tremendous energy, ISC division is suppressed, and the number of midgut epithelial cells decreases to reduce the energy cost for tissue maintenance under starved conditions [23,29,30,79]. Indeed, although ECs upregulate the expression of the cytokine *upd3* in response to starvation stress, ISCs do not activate proliferation due to the translational suppression caused by lack of S-adenosylmethionine [80]. The minimal proliferation of ISCs, which spares intestinal functions during prolonged nutrient scarcity, may be sustained by bypassing the requirement for amino acid sensing, as has been reported in larval growth [81,82]. Upon refeeding, the visceral muscle secretes *Drosophila* insulin-like peptide 3 (Dilp3) to activate ISC proliferation via insulin signalling (Figure 2c) [21,23,83,84]. EEs also support refeeding-dependent ISC proliferation by secreting Tachykinin, the neuropeptide that promotes *dilp3* expression in the visceral muscle [85]. Moreover, EBs sense food-derived mechanical stimuli, upregulate *upd3* expression via Yki, and thus also promote ISC division [86,87]. Collectively, the combination of ISC quiescence and niche-derived factors enables dynamic changes in stem cell activity during nutrient fluctuation.

Nutrient availability affects ISC division mode (symmetric or asymmetric) as well as their mitotic activity. Since the number of ISCs decreases during starvation, the ISC pool must be restored to achieve successful midgut resizing upon refeeding. Although 60–80% of ISC division is asymmetric in homoeostatic or starved conditions, refeeding increases the ratio of symmetric division such that ISC division generates two ISCs [20,23,83]. This asymmetric-to-symmetric shift drives ISC expansion and subsequent midgut growth. Mechanistically, starvation-induced JNK activation [20] and insulin signalling [83] promote the alteration in ISC division mode: phosphorylated JNK localizes at the mitotic spindle of ISCs and suppresses the cell cortex localization of Mud, a spindle regulator, to induce planar spindles, resulting in the symmetric outcome of ISC division [20,88]. How insulin signalling regulates the division mode of ISCs still remains elusive. One hypothesis suggests that insulin signalling interacts with JNK signalling to control spindle orientation [20].

## 4. Ploidy of differentiated cells

While stem cell proliferation plays a pivotal role in adult tissue plasticity, additional cellular mechanisms are also required to secure adaptive responses, especially in some contexts where functional stem cells are spatially and/or numerically limited [89–92]. Polyploidization is one mechanism by which differentiated cells regulate cell size and resistance to DNA damage across diverse species [93,94]. Indeed, polyploidization enables efficient wound repair, especially in genotoxic conditions; if *Drosophila* abdominal epithelial cells, most of which experience DNA damage during adult life, are forced to proliferate in lieu of polyploidization, wound healing is compromised due to mitotic errors [91,95]. The cell ploidy increases through endoreplication in which M phase or cytokinesis is skipped during the cell cycle. Recent studies have revealed the physiological importance of polyploidization in both regenerative and nutritional contexts.

### 4-1. Polyploidization during regeneration after tissue damage

**Figure 3.**
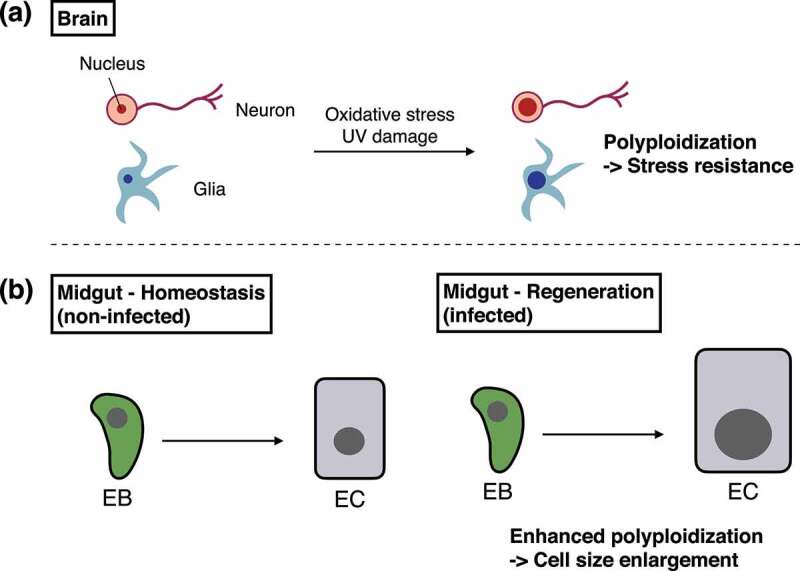
Polyploidization in differentiated cells.

The limited number of neuroblasts in the adult brain [35,36] may increase the demand for additional cellular mechanisms to ensure environmental adaptation. Consistent with this notion, Nandakumar et al.,recently found the role of polyploidization in protecting adult neurons and glia from cellular stresses [92]. While more than 98% of brain cells are diploid (2 N) just after eclosion, 10–20% become tetraploid or much higher ploidy until day 21. The polyploidization occurs both in neurons and glia throughout the brain, implying that polyploidy is correlated with brain plasticity during maturation and may potentially be involved in the maintenance of brain homoeostasis. Indeed, polyploid brain cells avoid cell death caused by UV irradiation, most likely because of their higher resistance to DNA damage (Figure 3a). Intriguingly, cells in the adult brain accelerate polyploidization in response to oxidative stress or UV damage [92]. These results suggest that neurons and glia can flexibly increase their ploidy to acquire stress resistance, which may minimize a need for neurogenesis under harmful conditions.

(a) In the adult brain, a subset of neurons and glia cells increase their ploidy in response to oxidative stress or UV damage. Polyploidization improves resistance against subsequent environmental stresses. (b) Polyploidization in EB-EC lineage is enhanced during regeneration in the adult midgut. Polyploidization promotes cell size enlargement of ECs, contributing to organismal survival upon pathogenic infection.

Studies using the adult midgut shed light on another role of polyploidization besides protection from DNA damage. In the adult midgut, endoreplication occurs in EB-EC lineage, resulting in polyploid ECs that can reach a maximum 128 N [79,96]. Inhibition of cell cycle progression in EBs shortens organismal survival upon pathogenic infection, indicating that endoreplication is essential for host defence together with ISC proliferation [96]. Mechanistically, the EGFR-Ras-MAPK cascade in newborn ECs accelerates cell size enlargement through polyploidization, promoting structural regeneration of the damaged midgut. Of note, mature ECs neither express EGFR protein nor activate the EGFR-Ras-MAPK cascade, suggesting that excessive overgrowth is prevented after regeneration [96]. These results suggest that the ploidy in the adult midgut contributes to regeneration through cell size regulation (Figure 3b). The two roles of polyploidy, cell size regulation and protection from DNA damage, are not mutually exclusive, and both functions ensure proper tissue regeneration.

### 4-2. Polyploidization in response to nutrient environment

Nutrient-absorptive ECs respond to nutrient environments, and thus the ploidy of ECs can contribute to the size regulation of the adult midgut. In contrast to the regeneration-associated polyploidization regulated by EGFR signalling, nutrient-rich diets promote the polyploidization of ECs via insulin-TOR signalling [79,97]. Given that insulin-TOR signalling also regulates ISC proliferation [23,85,90,98], one question is to what extent each cellular process contributes to midgut resizing. Bonfini et al., have addressed this question using two types of isocaloric diet: one high sugar diet, the other high in yeast (yeast being the primary source of protein and lipids in conventional fly food) [97]. They found that shifting from the high sugar diet to the high yeast diet promotes ISC proliferation, polyploidization in ECs, and the growth of the adult midgut. Unexpectedly, inhibition of ISC proliferation does not suppress midgut adaptive growth because of the augmented enlargement of ECs, which is mediated by TOR signalling-dependent polyploidization [97]. These results raise the possibility that the adult midgut senses the dysfunction of ISCs through mechanical tension and/or unknown cellular communication mechanisms to induce compensatory polyploidization of ECs. It would be interesting to know whether a similar compensatory response occurs during the midgut regrowth in response to starvation-refeeding cycle.

## 5. Cell fate plasticity

**Figure 4.**
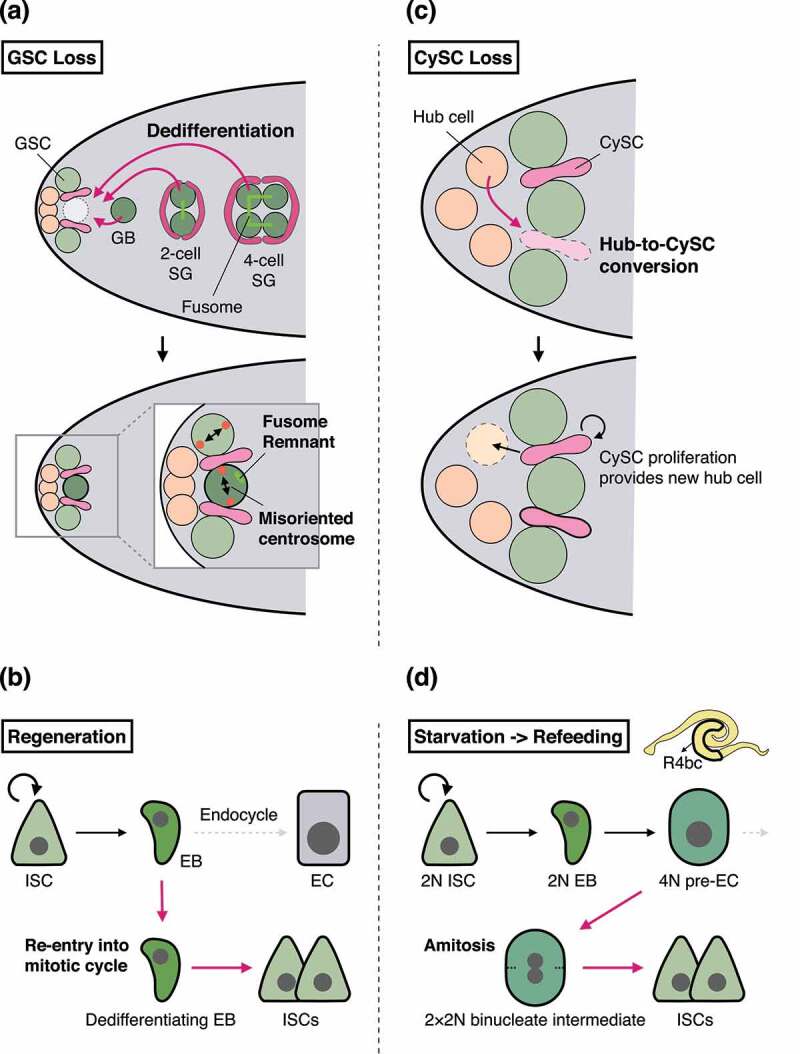
Cell fate plasticity in the male testis and the adult midgut.

Severe environmental challenges, such as irradiation or extrinsic toxins cause almost complete loss of tissue stem cells; however, the stem cell pool can be revived during the recovery phase [99,100]. But how do the adult tissues regain lost stem cells? Pioneering studies using the *Drosophila* adult gonad revealed that differentiating progenitor cells can revert to stem cell state upon genetic ablation of GSCs (Figure 4a) [101–104]. This cell fate plasticity, or dedifferentiation, revives functional GSCs which can generate the next generation. After these findings, the establishment of the lineage tracing technique has revealed the evolutionarily conserved process of dedifferentiation [99,105–109]. Accumulating evidence now suggests that dedifferentiation broadly occurs under conditions of stem cell loss, for instance, upon inflammation, injury, and starvation [29,45,105,110].

(a) In the male testis, GB, 2-cell SG, and 4-cell SG have a potential to dedifferentiate into GSC upon loss of GSCs. GSCs generated via dedifferentiation exhibit two morphological characteristics: misoriented centrosomes (neither centrosome is positioned close to the hub-GSC junction) and fusome remnant(s). (b) In homoeostatic conditions, EBs in the adult midgut exit from the mitotic cell cycle and instead undergo endoreplication, leading to terminal differentiation into polyploid ECs. During regeneration, however, a subset of EBs re-enter mitosis, resulting in dedifferentiation into ISCs. (c) Loss of CySCs induces conversion of hub cells into CySCs. Hub cells are then newly generated via CySC division. (d) In the midgut R4b-R4c region, tetraploid (4 N) pre-ECs undergo amitosis in response to refeeding after starvation. During amitosis, 4 N cells split into two cells through a binucleate intermediate (2 × 2 N) and generate new functional ISCs.

### 5-1. Cell fate plasticity during regeneration after tissue damage

The simple structure of the *Drosophila* adult midgut enables detailed observation of cellular dynamics during dedifferentiation. A recent study by Tian et al. has reported that EBs dedifferentiate into ISCs during regeneration after pathogenic infection (Figure 4b) [111]. While EBs lose mitotic capacity and differentiate into ECs in homoeostatic conditions [6,8,51,112], a subset of EBs re-enter M phase and generate daughter cells in response to infection. Surprisingly, EBs in M phase do not express ISC markers, and EB-derived daughter cells rather express ISC markers and exhibit multipotency. These results suggest that progenitor cells re-enter the mitotic cell cycle before dedifferentiation to generate two functional stem cells, providing a novel insight into the nature of cell fate plasticity.

In the adult gonad, cell fate conversion in response to GSC ablation accompanies characteristic morphological changes in dedifferentiating cells (Figure 4a), allowing detection of the dedifferentiation without lineage tracing [101,102]. This advantage makes the adult gonad a useful system to genetically address the molecular mechanisms of dedifferentiation. Indeed, Enhancer of Zeste [E(z)], a component of polycomb repressive complex 2, was identified as a regulator of dedifferentiation in the male GSC lineage [113]. In addition to this cell intrinsic mechanism, a non-autonomous mechanism is also reported: a transmembrane aminopeptidase, CG46339/Slamdance, functions in the hub cells to induce dedifferentiation of GSC daughters [114]. Although the exact mechanisms by which CG46339 regulate dedifferentiation remains elusive, CG46339 may target membrane protein(s) on adjacent GSCs or dedifferentiating cells, enabling spatial control of cell fate conversion.

While hub cells function as a niche that regulates dedifferentiation of GSCs [114], hub cells themselves can exhibit cell fate plasticity: hub cells convert into somatic stem cells (CySCs) upon their genetic ablation (Figure 4c) [115–118]. This lineage relationship is consistent with the fact that both hub cells and CySCs are generated from the same progenitor cells during development [119–121]. In the adult stage, hub cells become quiescent, but CySCs maintain mitotic activity; however, hub-specific overexpression of Cyclin D together with Cdk4 induces the hub-to-CySC conversion, indicating that re-entry into cell cycle is sufficient for the cell fate conversion [117]. Intriguingly, while hub cells maintain the stemness of CySCs, CySCs in turn maintain quiescence of hub cells by secreting Follistatin, which suppresses Activin signalling in hub cells [116,122,123]. These findings together suggest that the male testis is equipped with sophisticated mechanisms that precisely control the timing of cell fate conversion.

### 5-2. Cell fate plasticity during starvation-refeeding cycle

Although the physiological importance of cell fate plasticity remains largely unknown both in fly and mammals, studies using the *Drosophila* testis have provided some insights. The number of GSCs decreases during chronic protein starvation but recovers in response to refeeding [30,31,44,45]. This refeeding-dependent GSC recovery is delayed by blocking dedifferentiation of GSC daughters [45]. Moreover, mating increases the demand for dedifferentiation by causing exhaustion of pre-existing GSCs. As a result, failure in dedifferentiation leads to shrinkage of the testis and impaired spermatogenesis after repeated cycles of starvation, refeeding, and mating. These results indicate an essential role of dedifferentiation in maintaining tissue functions under stressful conditions [45].

The same study also revealed a striking feature of dedifferentiation: GSCs derived from dedifferentiation exhibit higher mitotic activity than pre-existing GSCs [45]. This finding is consistent with the notion that GSC exhaustion increases the demand for dedifferentiation [104,124]. Mechanistically, starvation-induced JNK activation, which drives cell fate plasticity in GSC daughters, is a candidate for providing the active proliferation capacity of GSCs. It is noteworthy that in homoeostatic conditions dedifferentiation is prevented by the RNA-binding protein me31B [125]. Given the link between dedifferentiation and tumorigenesis [126], dysregulation of dedifferentiation would lead to the highly proliferative nature of GSCs and eventually contribute to pathogenesis.

In addition to the male testis, nutrient stress stimulates cell fate plasticity in the adult midgut. Lucchetta and Ohlstein found that tetraploid EBs/ECs undergo ploidy reduction to generate new ISCs in response to refeeding after starvation (Figure 4d) [29]. In this process, tetraploid pre-ECs become binuclear (i.e. two diploid nuclei in one cell) without the formation of the spindle apparatus. The binuclear cell is then resolved into two daughter cells without the formation of the anillin-rich contractile ring. These cellular behaviours, which diverge from mitotic cell division, are equivalent to amitosis, an evolutionary conserved process initially described by Remak in 1841 [127–131]. While amitosis of pre-ECs can generate functional ISCs, it can cause loss of heterogeneity (LOH) due to the random segregation of chromosomes, which is a stark contrast to the fidelity of mitotic chromosome segregation. As a result of LOH, a deleterious mutation becomes homozygous, leading to disruption of tissue homoeostasis as exemplified by *kuzbanian* mutation, which induces the ISC differentiation defect [29]. Therefore, amitosis may function as a double-edged sword that revives lost stem cells but simultaneously bears potential pathological consequences. Of note, amitosis is observed only in the R4b-R4c region of the posterior midgut where the number of ISCs is most strongly decreased (~80% reduction) by starvation [29,132,133]. These results raise the possibility that amitosis occurs only in extremely severe conditions. Indeed, amitosis does not occur during the nutrient-dependent midgut growth after eclosion, but it is induced when ISC proliferation is blocked by a microtubule depolymerizing drug. Collectively, these results imply that amitosis is a backup mechanism to compensate for ISC loss or dysfunction.

## 6. Conclusion and future perspective

Adult tissues in *Drosophila* share common cellular processes (stem cell division, polyploidization, and cell fate conversion) to exert their plasticity against environmental challenges. The combination of these cellular responses promotes rapid adaptation to environmental fluctuations and also secures adaptation by enabling compensatory responses. In addition to their physiological importance, studies using *Drosophila* models have unveiled molecular players that regulate the cellular mechanisms underlying adult tissue plasticity and have identified the niche factors that coordinate different cell type behaviours and thus orchestrate environmental responses throughout the tissue. Given the evolutionary conservation of cellular processes, signalling pathways, and molecules discussed here, findings in *Drosophila* will continue to provide clues to understand environmental responses in different animal species, including humans.

One of the fundamental questions that remains unanswered is the origin of new stem or progenitor cells, which are generated during regeneration and nutrient adaptation. Although two cellular processes, dedifferentiation and symmetric division of pre-existing stem cells, are involved in stem cell expansion, their relative contributions are largely unknown outside of the case of stem cell ablation wherein all pre-existing stem cells are lost [101,102,134]. However, given that complete loss of stem cells is extremely rare in physiological contexts, comprehensive investigations are required in future research. Specifically, for instance, does the combination of symmetric ISC division and amitosis of ECs fully explain the ISC expansion during starvation-refeeding cycle in the adult midgut? Although amitosis occurs only in the R4b-R4c regions [29], other regions may utilize different strategies to increase the ISC pool. Supporting this possibility, in the male testis, dedifferentiation occurs under the condition of ~30% loss of GSCs [30,31,45]. Given that a much greater reduction in the number of ISCs (30–80% decline) can occur in the midgut during starvation, either dedifferentiation or an unidentified cellular phenomenon may play a role in ISC expansion [23,29–31]. Recent studies have revealed that lineage commitment in the midgut epithelium can be altered by genetic depletion of transcriptional regulators, such as *Klumpfuss, Lozenge*, and *Tramtrack*, further supporting the possibility of unprecedented cell fate plasticity [51,112,135]. Similarly, how does the number of adult neuroblasts dramatically increase upon brain injury? During regeneration of the adult brain, 50–100 mitotic clones are induced in the central brain, while the number of potential neuroblasts is only 0–10 in the homoeostatic condition [35,65]. Future investigations must address whether the pre-existing neuroblasts are sufficient for the regeneration or if other cell types can revert to neuroblasts in response to severe damage.

Lineage tracing and histological observations using *Drosophila* adult tissues have shown that only a subset of specific cell types can respond to environmental fluctuation. In fact, neurons that acquire polyploidy constitute 10–20% of all neurons [92]. Endocycling cell populations have also been identified in the regenerating mammalian kidney, a cell population that could be conserved in the *Drosophila* Malpighian tubules where at least three subtypes of polyploid cell exist [136,137]. To identify so specialized a subpopulation, rapidly evolving single cell ‘omics’ approaches are powerful and game-changing tools, and, indeed, the single-cell transcriptome atlas has already been established for most adult tissues in homoeostatic conditions [72,138,139]. By comparing single cell states during environmental fluctuations with those in homoeostatic conditions, we can identify novel subpopulations and the intercellular relationships that coordinate environmental responses. With single cell approaches together with sophisticated genetic tools and careful histological observations, *Drosophila* adult models will further contribute to a better understanding of the mechanisms and roles of adult tissue plasticity.

## Data Availability

No datasets were generated or analyzed during the current study.
